# Brooding duration does not depend on cat predation risk but is related to weather and phenology in the wandering albatross (*Diomedea exulans*)

**DOI:** 10.1002/ece3.70174

**Published:** 2024-08-29

**Authors:** Charlotte Bourgoin, Christophe Barbraud, Tobie Getti, Karine Delord, Frédéric Angelier, Aymeric Bodin, Pierrick Blanchard

**Affiliations:** ^1^ Centre de Recherche sur la Biodiversité et l'Environnement (CRBE) Université de Toulouse, CNRS, IRD, Toulouse INP, Université Toulouse 3 – Paul Sabatier (UT3) Toulouse France; ^2^ Centre d'Études Biologiques de Chizé CNRS – La Rochelle Université, UMR 7372 Villiers‐en‐Bois France; ^3^ Réserve Naturelle Nationale des Terres Australes Françaises TAAF Saint‐Pierre France

**Keywords:** brooding, feral cats, Kerguelen archipelago, parental investment, predator–prey relationships, wandering albatross

## Abstract

Parental investment increases offspring fitness at the expense of the parent's ability to invest in other offspring. In many animal species, parents guard their offspring after birth. The parental decision over the duration of this period is expected to be triggered by the associated fitness costs and benefits for both offspring and parents. Here, we evaluated the relevance of several intrinsic and environmental variables in determining brooding period duration in the wandering albatross (*Diomedea exulans*) and questioned whether brooding duration was related to chick subsequent survival and biometry prior to fledging. We used a semi‐experimental design to increase the variance in cat abundance, a recent predator of albatross chicks, and predicted that an increased predation risk at the nest scale would trigger longer chick brooding and thus, protection. In addition, we questioned the influence of weather conditions, hatching date, and characteristics of chicks (sex and biometry) and parents (sex and age) on brooding duration. We report no effect of predation risk or parental characteristics on brooding duration. However, the probability for a parent to end brooding decreased with forthcoming unfavorable weather. Our data also revealed reduced brooding duration for late‐hatched chicks and a positive association between brooding duration and chick structural size, and between the frequency of shifts between parents and chick structural size. Finally, brooding duration was not associated with chick survival or with chick biometry prior to fledging. We discuss these results in light of pre‐existing hypotheses on fitness costs and benefits associated with brooding duration for chicks and parents.

## Graphical abstract

In many species, parents take care of their offspring after birth or hatching and face a trade‐off between the costs and benefits associated with the duration of this “guarding period”. Here, we investigated brooding duration variability in a colony of wandering albatrosses experimentally divided into zones with and without feral cat control, a novel predator of albatross chicks in Kerguelen. We report no effect of predation risk on brooding duration but the probability for a parent to end brooding decreased with forthcoming unfavorable weather, and late‐hatched chicks were less brooded. We also show a positive association between brooding duration and chick size, and between the frequency of shifts between parents and chick size. We discuss these results in light of our system specificities and of pre‐existing hypotheses on costs and benefits associated with brooding duration experienced by chicks and parents.

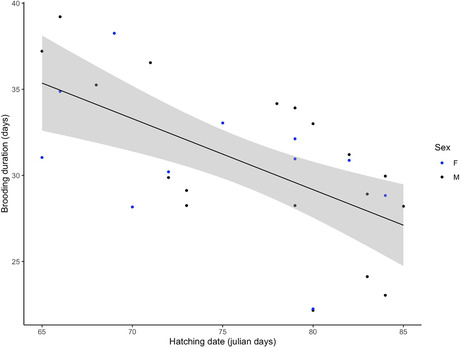

## 1 INTRODUCTION

Parental care refers to any non‐genetic contribution by an adult whose function is to increase the fitness of its offspring (Royle et al., 2012). It encompasses a wide range of behavioral, morphological, and physiological traits and occurs not only in many animal taxa, from invertebrates to vertebrates (Royle et al., 2012), but also in plants (Lacey & Herr, 2005). When the parental contribution increases offspring fitness at the expense of the parent's ability to invest in other, current or future, offspring, it is referred to as parental investment (Trivers, 1972). Numerous studies have investigated the factors shaping individual decisions of parents facing such an expected trade‐off. In white‐tailed deer (*Odocoileus virginianus*), Therrien et al. (2007) showed that an experimental food restriction of 20% did not affect mass gain by reproductive females, while their fawn growth and survival decreased by 26% and 35%, respectively, suggesting that females adopted a conservative strategy that favors their reproductive potential over their current reproduction. In adult great tits (*Parus major*), experimentally enlarged clutches translated into decreased parasite resistance and survival in first‐year breeding females, more sensitive to harsh environmental conditions (Stjernman et al., 2004).

In many invertebrate and vertebrate species, parents take care of their offspring after birth or hatching, during a “guarding period” (Royle et al., 2012). In birds, published results show a large inter‐ and intra‐specific variability in brooding duration. For example, in grey‐headed albatrosses (*Thalassarche chrysostoma*), brooding duration ranges from 16 to 39 days (Catry et al., 2006) and from 2 to 8 days in snow petrels (*Pagodroma nivea*) (Tveraa & Christensen, 2002). The intraspecific variability in parental decision over brooding duration suggests the existence of a trade‐off at the individual scale between the fitness costs and benefits associated with ending or continuing brooding (Brodin et al., 2003; Catry et al., 2006, 2009, 2010; Durant et al., 2004; Lewis et al., 2004; Rothenbach & Kelly, 2012; Tveraa et al., 1998; Tveraa & Christensen, 2002; Varpe et al., 2004). Staying at the nest enables parents to protect chicks against predation, unattended chicks being more likely to be predated (e.g., Rothenbach & Kelly, 2012), or against attacks by conspecifics (Lewis et al., 2004). The continuous presence of a parent at the nest also provides protection from adverse weather conditions, including cold (Visser & Ricklefs, 1993), sun and rain (Johnson & Best, 1982), or storms (Weathers et al., 2000). Furthermore, it allows the chick to be fed with regular small meals, that is, in a way adapted to its limited gut capacities and rapid digestion (Awkerman et al., 2005; Catry et al., 2009). Finally, the presence of a parent reduces stress sensitivity (Dupont et al., 2021) and promotes behavioral development (Shimmura et al., 2010) of chicks. Altogether, these results may explain why chicks brooded for a longer period showed increased survival until fledging, although this pattern proved to depend on the considered year and species (Catry et al., 2006, 2009, 2010; Jones et al., 2014). However, the costs associated with an extended brooding period are also noticeable. During brooding, because parents fast, their condition often declines (e.g., Tveraa et al., 1998). Concomitantly, the chicks' energetic requirements are expected to increase as they age, triggering the necessity of food delivery by both parents and thus, mechanistically, the end of the brooding period (Brodin et al., 2003; Catry et al., 2006; Durant et al., 2004).

Here, we evaluated the relevance of several intrinsic and environmental variables as selective pressures that may trigger parental decision over brooding period duration in the wandering albatross (*Diomedea exulans*) and examined whether brooding duration was related to chick subsequent survival and biometry prior to fledging. We first focused on predation pressure at the nest scale and hypothesized that parents facing increased predation risk should display longer brooding periods in order to offer greater protection to their chick (Catry et al., 2009). In our study area, northern giant petrels (*Macronectes halli*) and feral cats (*Felis catus*), recently reported to prey on wandering albatross chicks (Barbraud et al., 2021; Blanchard et al., 2024), are their only known predators, and increased brooding duration has been suggested as a potential parental tactic to minimize predation risk (Blanchard et al., 2024; Dilley et al., 2013). Here, we experimentally increased the inter‐nest variance in feral cat abundance (Blanchard et al., 2024), thereby allowing an appropriate investigation of the role of predation risk in this parental decision.

Besides directly affecting parental decision over brooding duration, predation risk was further suggested to trigger an effect of hatching date on brooding duration, in line with the “synchronization hypothesis” (Catry et al., 2009). This hypothesis suggests that a seasonal decline in guarding time will result in later‐born chicks being left unguarded at the same time as earlier‐born chicks. This in turn would favor a dilution (i.e., the larger the group, the lower the chance that one particular individual will be the one targeted) and/or satiation (i.e., the predator becomes satiated, or swamped, at a certain prey density) effect for the predator (Ims, 1990). We thus also questioned hatching date as a determinant of brooding duration in wandering albatrosses.

We then focused on weather as a potential determinant. Since unfavorable weather conditions, and in particular strong winds (Momberg et al., 2023) and cold exposure (Lefebvre, 1977), are detrimental to albatross chicks, and since birds have been shown to perceive atmospheric pressure and to behave accordingly (Breuner et al., 2013; Metcalfe et al., 2013), we hypothesized that parents should be less inclined to end brooding under current or forthcoming unfavorable weather conditions.

Some additional potential relevant variables in the context of brooding duration were also investigated: (i) chick biometry (i.e., size and condition), expected to relate not only to chicks energetic requirements but also to their ability to face harsh weather and predators and thus to trigger parental decision over brooding termination (Catry et al., 2006, 2009); (ii) the frequency of shifts between parents, a parameter relating both to chicks and parents quality (Lequette & Weimerskirch, 1990); and finally, (iii) chick sex and parental age and sex, previously shown to shape foraging and brooding patterns in wandering albatrosses parents (Jones et al., 2014; Weimerskirch et al., 2000; Weimerskirch & Lys, 2000).

Finally, we questioned whether brooding duration may in turn shape chicks' fitness. We investigated the relationship between brooding duration and subsequent chick mortality, while accounting for the main sources of mortality in the population and tested the relationship between brooding duration and chick biometry prior to fledging, reported to affect post‐fledging survival in this species (Weimerskirch et al., 2000).

## 2 MATERIALS AND METHODS

### 2.1 Study area

**FIGURE 1:**
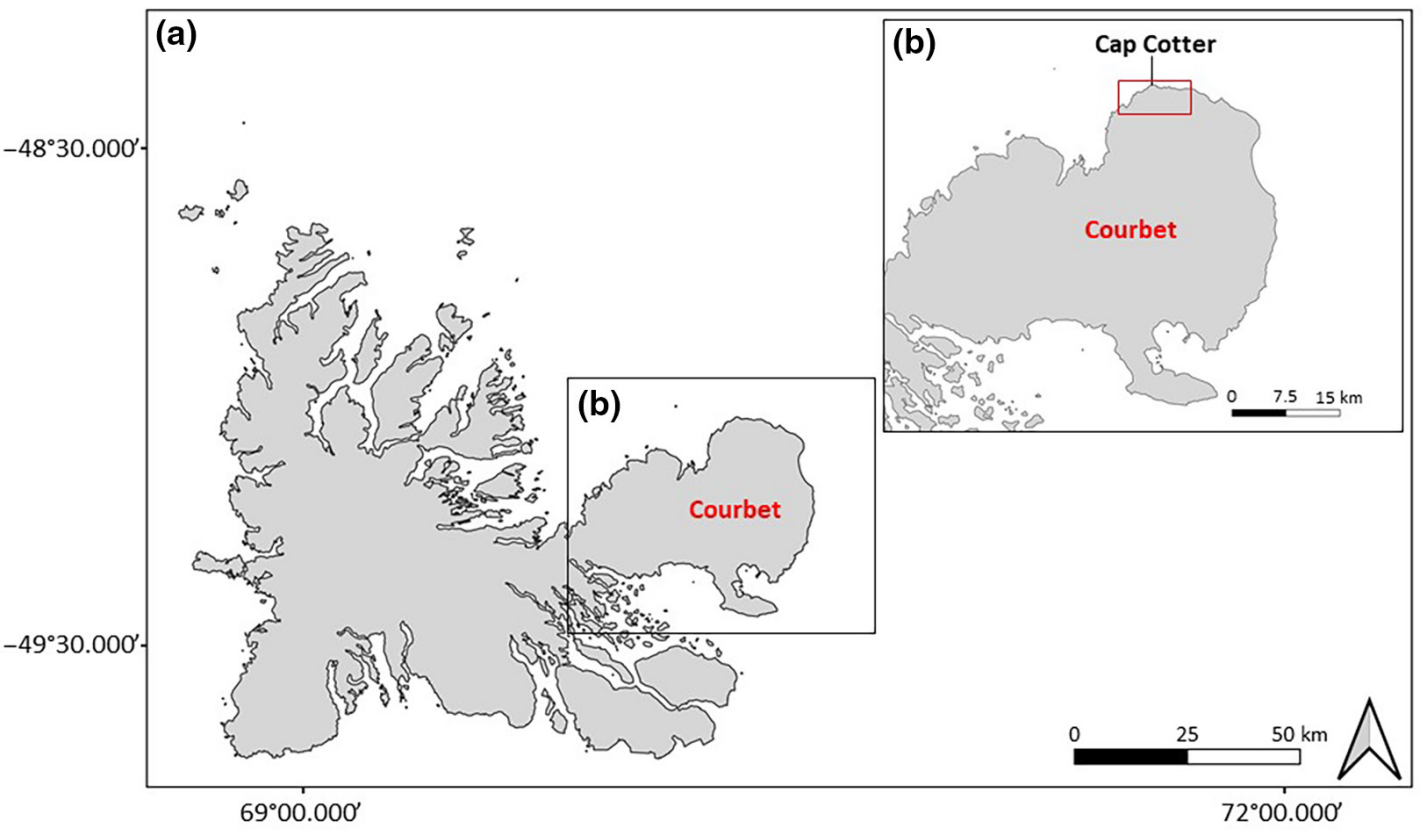
Overall map of Kerguelen (a) and Courbet peninsula (b). The red rectangle in panel (b) refers to our study area.

This study was carried out from December 2021 to October 2022 in the Kerguelen archipelago, southwestern Indian Ocean. Kerguelen includes a main island (“Grande Terre”, ~6700 km^2^) and hundreds of smaller islands (Figure 1a). Our study area was localized at Cap Cotter (49.057867° S, 70.304915° E) on the Courbet peninsula (Figure 1b), and covers an area of ~20 km^2^, dominated by the native herbaceous perennial *Acaena magellanica*.

### 2.2 Wandering albatross

From late December to early January, female albatrosses lay a single egg that will be incubated continuously for about 80 days, alternately by both parents (Weimerskirch et al., 1993). After hatching (around mid‐March), partners alternate chick brooding and short foraging trips at sea so that chicks are continuously brooded by one parent for about 1 month (Weimerskirch et al., 1993). The chick is then left alone and parents mix short and long trips to regularly feed their chick throughout winter and spring. Fledging occurs around late November. Sexual dimorphism in mass and size occurs both in adults and old chicks, with males being heavier and larger than females (adults: 9.6 and 7.8 kg, fledglings: 10.6 and 9.3 kg, for males and females, respectively, Weimerskirch et al., 2000).

Feral cats and giant petrels are the only known predators of wandering albatross chicks in Kerguelen (Blanchard et al., 2024). Unlike on Marion Island, for example, attacks by mice (Jones & Ryan, 2010) have, to our knowledge, never been recorded. However, as brown skuas (*Catharacta antarctica lonnbergi*) predate chicks of related albatross species in other localities (Catry et al., 2006; Forster & Phillips, 2009) and may be perceived as a predation risk by wandering albatrosses in Kerguelen (authors, pers. obs), we also considered this species in the analyses questioning the effect of predation risk on parental decision.

### 2.3 Cat control design

In order to increase the inter‐nest variance in cat abundance, the studied colony was divided into four experimental zones of 2 km^2^: two zones with feral cat control and two zones without. We chose zones with the same kind of habitats and alternated controlled and uncontrolled treatments in order to reduce the probability for an unidentified environmental confounding factor to occur. To control the cat population, we used double‐door traps (approximately 0.2 km^2^ per trap, as reported in Barbraud et al., 2021), leg‐hold traps, and shooting with riffle Tikka T3x 222. Overall, six cats were killed during the control (February 26–March 6, i.e., just before the brooding period started). As zones were defined in similar habitats, cat detection probability was considered to be the same in each zone, allowing calculation of a kilometric index (KI) to assess cat abundance (Vincent et al., 1991). Hence, while the abundance of cats was not significantly related to zone identity before the cat control occurred, significantly fewer cats were observed in controlled zones (0.04 and 0.02 cats/km) than in uncontrolled zones (0.19 and 0.30 cats/km) after the control (Blanchard et al., 2024). More details related to cat control can be found in Blanchard et al. (2024).

### 2.4 Data collection

The datasets analyzed during the current study are available in the figshare repository, at https://figshare.com/s/75570849ccf548961ed8. All experimental procedures were approved by the Ministère de l'Enseignement Supérieur, de la Recherche et de l'Innovation (permit APAFIS#31386–2021042717283578).

#### 2.4.1 Brooding duration, shifts frequency, and predation variables: The use of camera traps

In our study area, 73 eggs were laid, of which 57 chicks hatched. For each of these chicks, except one, the exact date of hatching was determined by checking nests every 2 days around the expected date (Blanchard et al., 2024). A camera trap (Reconyx HP2X or PC 900) was positioned in front of 41 of these chicks. Camera traps were positioned 8 m from the nest, 1 m above ground, and fixed on aluminum poles with pieces of orange adhesive tape to facilitate detection by flying birds (Blanchard et al., 2024). The camera traps were set up to take a picture every 2 min, 24 h per day, starting 2 days after hatching for logistical reasons, up to the end of July, thereby largely including the 1‐month brooding period. Of these 41 monitored nests, 30 chicks reached the end of brooding (Blanchard et al., 2024). Some of the photos (0–10.63% of photos per nest) were unusable due to fog, snow, or because the camera had been destroyed by reindeer (*Rangifer tarandus*) or elephant seals (*Mirounga leonina*).

A total of 631,442 photos were individually analyzed in order to determine brooding duration, number of parental shifts, and number of known (cats and giant petrels) or potential (skuas) predators visible on each picture, as detailed in the following paragraphs.

Brooding duration was defined as the number of days during which a parent was continuously on, or in the immediate vicinity of the chick until the chick was left alone for a minimum of 12 consecutive hours. It was calculated by the difference between the day when brooding ended (rounded to the nearest hour) and the hatching day (time arbitrarily set at midday).

An index of predation risk by birds (giant petrels and skuas were considered together to reduce the number of variables, thereafter “predator birds abundance”) and by cats (thereafter “cat abundance”) at the nest scale was calculated by dividing the total number of predators of each type (birds or cats) identified on the photos (including flying birds) taken during the entire brooding period by the total number of photos taken during the same period, excluding unusable photos (e.g., snow on the lens). Predator distance was not considered. An index of overall predation risk (incorporating both birds and cats, thereafter “total predator abundance”) was also calculated. Given the distributions of the three predation‐related variables, we used categorical variables, with 0 for low predator abundance (i.e., with an abundance lower than the median for “predator birds abundance” and for “total predator abundance” and with an abundance of 0 cat observed for “cat abundance”) and 1 for high predator abundance.

The frequency of shifts between parents (individually recognizable on the photos) was calculated by dividing the number of recorded shifts by the total duration of the brooding period, minus the first 2 days of brooding without a camera. We also calculated the proportion of time spent at the nest by each of the two parents and recorded the sex of the parent ending brooding.

Finally, the exact age of the parents that had been ringed as chicks (20 females and 16 males) was known, ranging from 8 to 42 years old.

#### 2.4.2 Chick biometry and sex: Capture

All alive chicks were captured between 30 and 33 days after hatching (*n* = 45; mean age = 30.3 days; measured 7–26 April), that is, at the expected average chick age at the end of brooding (Weimerskirch et al., 1993). The lengths of culmen, head (from the occipital bone to the extremity of the beak), both tarsi, as well as maximum bill height and head width were measured with a caliper (±0.1 mm), and the lengths of both wings with a ruler (±1 mm). Finally, the chicks were weighed to the nearest 50 g using a Salter scale. To obtain the structural size and the condition of each chick, we (1) performed a PCA on standardized measures and considered the scores on the first axis (84.97% of total inertia) as an index of structural size of the chicks (a high score corresponding to a large chick), and then (2) regressed the chick mass by this index and considered the residuals as an index of body condition (see Blanchard et al., 2007, for the same approach for the same species). Finally, a blood sample was collected from the brachial vein of each chick, which allowed molecular sexing following procedure detailed in Blanchard et al. (2007).

On October 7 and 8, that is, about 2 months before fledging, the surviving chicks were captured (*n* = 21). Their condition and structural size were measured following the same procedure. This allowed us to investigate the relationships between brooding duration and chicks' biometry prior to fledging.

#### 2.4.3 Chick survival

The camera trap monitoring was conducted until the end of July, allowing us to confidently assess the causes of death for all but one chick. Identified causes of death were meteorological reasons (42.4%), predation by cats (24.2%) or giant petrels (6.1%), or poor parental care (18.2%) (details in Blanchard et al., 2024). Three chicks died between the end of July and the last capture in October. For these chicks, we had no clue about their cause of death.

#### 2.4.4 Weather

The mean wind speed and the average air temperature were measured every hour at a weather station located in the center of the study colony. We then calculated the Wind Chill Index (thereafter, the WCI) for each hour, as:

WCI = 13.12 + 0.6215 × 𝑇𝑎 − 11.37 × 𝑉^0,16^ + 0.3965 × 𝑇𝑎 × 𝑉^0,16^, with Ta, the air temperature in°C and *V*, the wind speed in km h^−1^ (Osczevski & Bluestein, 2005). This index has already been used for the black‐browed albatross *Thalassarche melanophris* in the same theoretical context (e.g., Catry et al., 2010).

### 2.5 Statistical analyses

Analyses were performed using R, version 4.2.3 (R Core Team, 2022). We followed the same following procedure in all analyses, except for the survival analysis, described below. The significance of each term (variable or interaction) was tested by building a model with and a model without this term and by comparing the deviances between both models with an *F*‐test (in case of a continuous dependent variable, e.g., brooding duration) or an χ^2^ test (in case of a binary dependent variable, e.g., presence/absence of a parent) with the appropriate number of degrees of freedom. When the interaction was not significant, it was removed from the model before testing the main terms. The main terms were always kept in the model when testing their interaction. Assumptions (overdispersion, homoscedasticity, and normality of models' residuals) were checked with packages *DHARMa* (Hartig, 2022) and *car* (Fox & Weisberg, 2019).

#### 2.5.1 Brooding duration as a dependent variable

Before investigating the relevance of the intrinsic and environmental variables in determining brooding period duration, we questioned whether these independent variables were related using correlations, Student's, or χ^2^ tests depending on the type of considered variables. In order to avoid increasing the number of considered models and given our relatively small sample size, we then built models with brooding duration as the dependent variable and (1) only one independent variable, (2) two independent variables with their interaction when biologically expected (i.e., the interactions between chick's biometry and predation risk, see below), and (3) two independent variables when significantly related, without their interaction. In the latter case, we checked whether the variance inflation factor was <2. In one case of strong collinearity (variance inflation factor > 3), we also tested each independent variable independently and found no significant result.

To test for the effect of WCI on the probability for the parent to end brooding, we considered the day of departure (variable “departure” = 1) and the 5 days before the day of departure (variable “departure” = 0) and, for each of these 6 days, calculated the WCI of the day and of the following day. We then investigated the effect of these two WCI‐related variables on the probability of departure with a generalized mixed model with nest identity as a random term, using package glmmTMB (Brooks et al., 2017). To avoid increasing the number of models, we only considered the effect of the size and the condition of the chick in these models. We only tested their interaction with the WCI‐related variable, and not their main effect, as chicks were captured only once, and not each of the six considered days.

Finally, we questioned whether parental sex impacted the total amount of time spent brooding and the decision to end brooding using a one‐sample Student's test and an exact binomial test, respectively.

#### 2.5.2 Brooding duration as an independent variable

Here, we questioned whether brooding duration explained the variability in chicks' biometry 2 months before fledging, that is, at second capture. We included an interaction with chick sex in the models and followed the same procedure as previously described.

#### 2.5.3 Survival analysis

The observations of live and dead chicks formed a set of observable events from which we estimated the proportion of chicks that died by specific causes identified from the visualization of photos from camera traps. Briefly, we used a probabilistic capture–recapture multi‐event model that linked the observed events to transitions between possible alternative individual states (Schaub & Pradel, 2004). We considered chicks can move across six states: alive (A, *n* = 24), death from predation by cats (DC, *n* = 8), death from predation by giant petrels (DP, *n* = 2), death by flooding (DF, *n* = 11), death from inadequate parental care (DA, *n* = 6), and death from other causes (DU, *n* = 6) (see Blanchard et al., 2024). We also included an additional state that corresponds to an unobservable dead state (Tavecchia et al., 2012). Using photos from the camera traps, chicks were observed in seven mutually exclusive events. The first event (coded 0) is a non‐encounter and indicates that the chick was not observed. This corresponded to the observation period before the chick was born and after its death. Events 1, 2, 3, 4, 5, and 6 refer to observations of chicks in the A, DC, DP, DF, DA, and DU states, respectively. Details of the model parameterization and selection can be found in Blanchard et al. (2024). We then tested for the effect of brooding duration (considered as an individual covariate) on chick survival probability, on the probability of being predated by a cat, on the probability of being predated by a giant petrel, and on the probability of dying from flooding.

## 3 RESULTS

Brooding period duration ranged from 22.2 to 39.2 days (mean ± SD: 30.7 ± 4.2 days; *n* = 30). Overall, females tended to spend more time brooding than males (53.25% of the brooding time, *t* = 1.79, df = 29, *p* = .08) and both sexes ended brooding in the same proportions (11 females and 19 males, i.e., number of trials = 30, *p* = .20).

### 3.1 Brooding duration as a dependent variable

We first investigated the relationships between the independent variables. Four pairs of independent variables were significantly, or nearly significantly (i.e., 0.05 < *p* < .10), related: the frequency of shifts during brooding and the structural size of the chick at first capture (*t* = 4.27, df = 24, *p* < .001), the “predator birds abundance” and the “total predator abundance” (χ^2^ = 18.23, df = 1, *p* < .001), paternal age and the “total predator abundance” (mean paternal age of 17.75 years for low abundance and 13.38 years for high abundance; *t* = 1.92, df = 13.01, *p* = .08), and the frequency of shifts during brooding and the “cat abundance” (mean frequency of shifts of 0.29 for low abundance and 0.24 for high abundance; *t* = 1.98, df = 16.96, *p* = .064). Among the above relationships, besides those expected by construction (e.g., “predator birds abundance” and “total predator abundance”) or without any obvious biological reason (e.g., paternal age and “total predator abundance”), the positive relationship between the frequency of shifts during brooding and the structural size of the chick at first capture might be biologically relevant as we expect chicks receiving more food to grow faster. However, the frequency of shifts considered here was calculated over the whole brooding period, that is, well after the first capture, and thus the measure of the structural size for some chicks. Hence, to explore the relationship between the frequency of shifts and structural size more closely, we focused on chicks still brooded when captured and also found a positive relation between the frequency of shifts from hatching to first capture and the chick structural size at first capture (*n* = 15, *F*_1,13_ = 5.83, *p* = .03).

**FIGURE 2:**
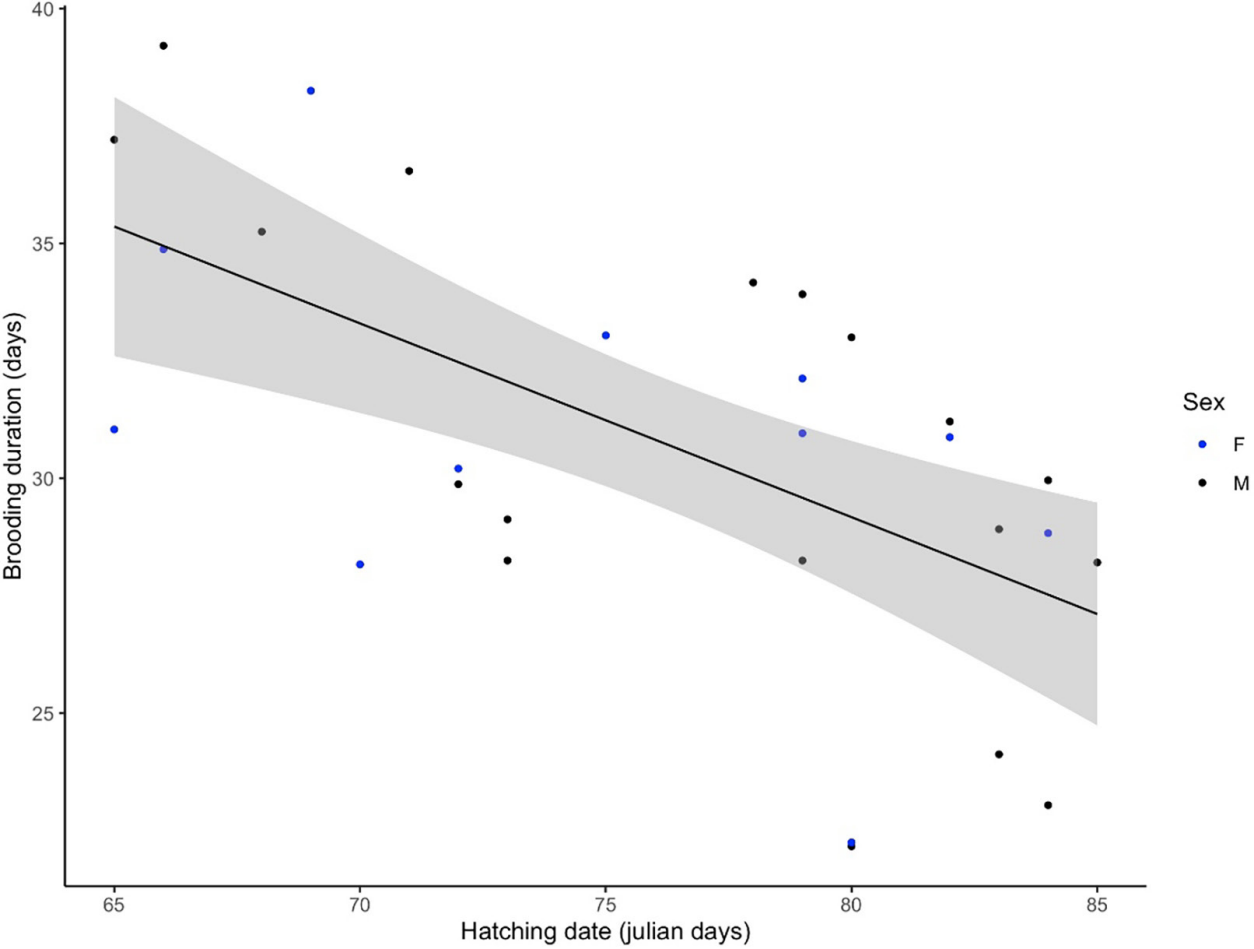
Brooding duration in relation to chick hatching date and sex (*F*_1,28_ = 15.41, *p* < .001). Hatching dates are Julian days, with day 65 = March 6, 2022.

**FIGURE 3:**
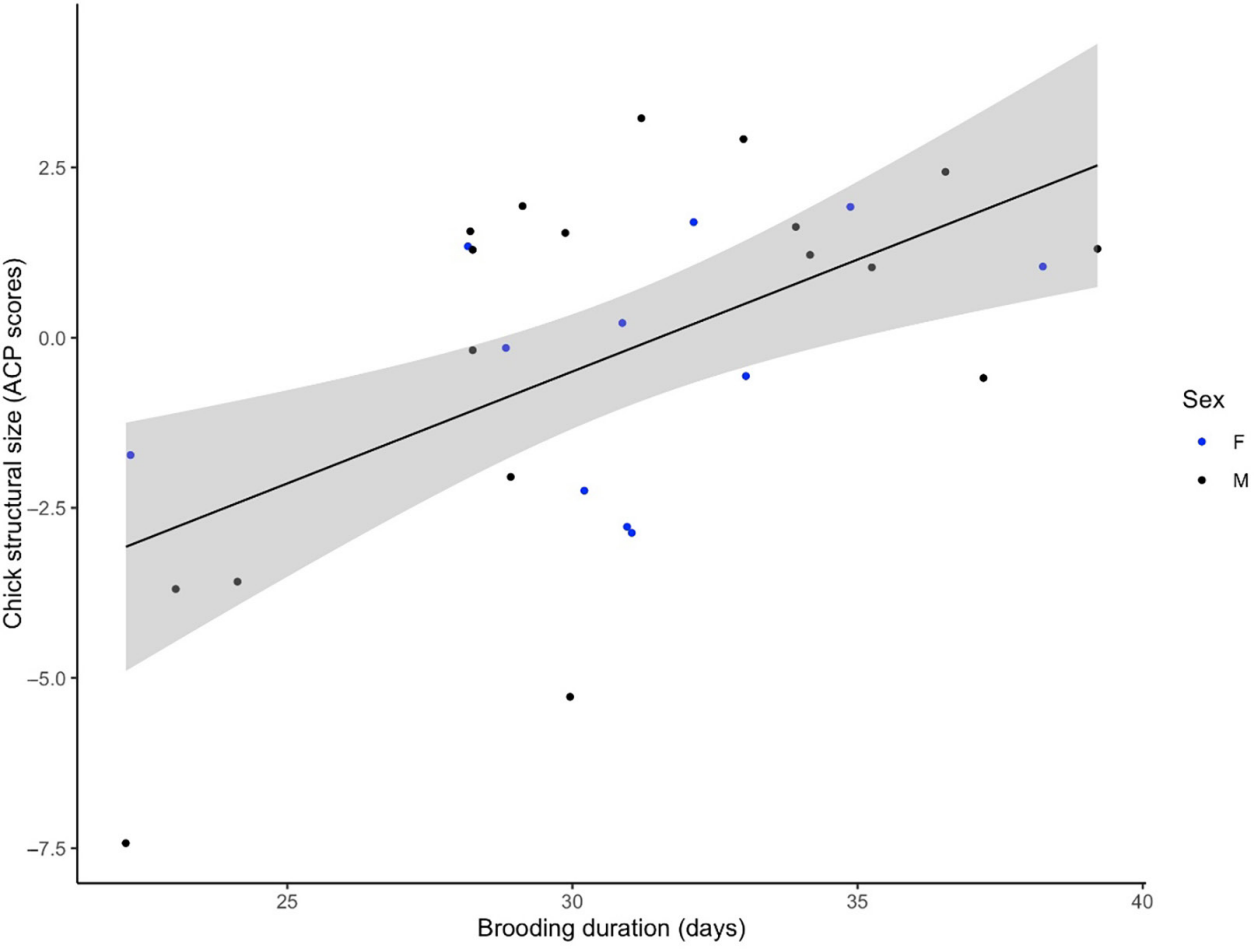
Chick structural size in relation to brooding duration and chick sex (*F*_1,27_ = 6.30, *p* = .02). Structural size is the chick's score on the first axis of a PCA including lengths of culmen, head, both tarsi, maximum bill height, and both wings of all chicks, captured at 30–33 days old.

We then questioned which of the considered independent variables were significantly related to brooding duration and retained hatching date and structural size at first capture (Table 1). Chicks that hatched earlier underwent longer brooding (*F*_1,28_ = 15.41, *p* < .001, Figure 2) and chick structural size at first capture was positively related to brooding duration (*F*_1,27_ = 6.30, *p* = .02, Figure 3). As first capture occurred on average at 30.3 days for all chicks and brooding duration varied from 22 to 39 days, the latter relationship indicates that smaller chicks at capture had been less brooded while bigger chicks at capture were further brooded after capture. The other investigated variables were not significantly related to brooding duration (Table 1).

**TABLE 1:** Tests of the relationships between brooding duration and the considered intrinsic and environmental biological variables.

Variable or interaction	*F*	df	*p*
**Hatching date**	**15.41**	**1,27**	**<.01**
**Chick structural size**	**>6.30**	**1,27**	**<.02**
Shifts frequency	<2.83	1,27	>.10
Cat abundance	1.62	1,27	.22
Predator birds abundance	0.41	1,27	.53
Total predator abundance	<1.67	1,27	>.21
Chick condition	<0.02	1,27	>.88
Chick sex	0.03	1,27	.88
Paternal age	1.67	1,13	.22
Maternal age	0.02	1,17	.88
Chick structural size: Total predator abundance	0.01	1,26	.95
Chick condition: Total predator abundance	0.42	1,26	.52

*Note*: Variables in bold are significantly related to brooding duration. When a variable is included in several different models (see text), we present the maximum *p*‐value in case of a significant variable and the minimum *p*‐value in case of a non‐significant variable. Sample sizes for paternal and maternal age are reduced as some adult birds had unknown ages.

**FIGURE 4:**
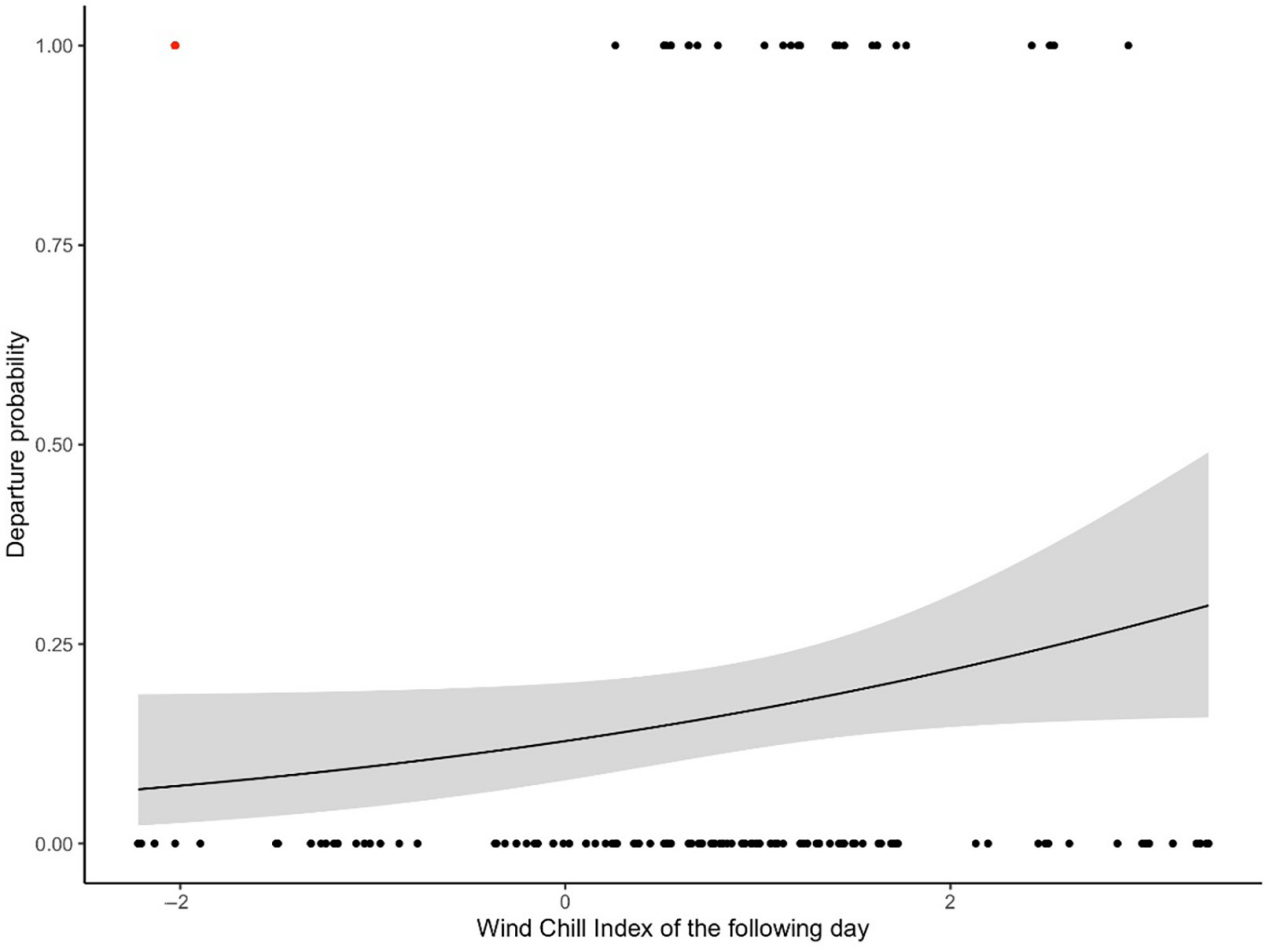
Departure probability of the parent in relation to the wind chill index (WCI) of the following day (χ^2^ = 3.87, df = 1, *p* = .049; when the red dot is removed, *p* = .01).

Finally, the probability for a parent to end brooding was significantly and positively related to the WCI the following day (χ^2^ = 3.87, df = 1, *p* = .049, Figure 4), indicating that parents were less inclined to end brooding in case of unfavorable weather the following day. When one data point (red dot in Figure 4) was removed, the *p*‐value decreased to .01. The other investigated variables or interactions were not significant (Table 2).

**TABLE 2:** Tests of the relationships between the probability for a parent to end brooding and the considered variables.

Variable or interaction	χ^2^	df	*p*
**WCI (day + 1)**	**3.87**	**1**	**.049**
WCI (day)	0.39	1	.53
WCI (day): Chick structural size	0.37	1	.55
WCI (day + 1): Chick structural size	3.45	1	.06
WCI (day): Chick condition	0.08	1	.78
WCI (day + 1): Chick condition	1.40	1	.24

*Note*: Variables in bold are significantly related to brooding duration. WCI for wind chill index. When the red dot in Figure 4 is removed, the *p*‐value associated with the WCI (day + 1) effect decreased to 0.01.

### 3.2 Brooding duration as an independent variable

Brooding duration did not explain the variability either in chick structural size (in interaction with sex: *F*_1,10_ = 0.04, *p* = .85; alone: *F*_1,11_ = 1.13, *p* = .31) or condition (in interaction with sex: *F*_1,10_ = 0.58, *p* = .46; alone: *F*_1,11_ < 0.01, *p* = .97) prior to fledging, that is, at second capture. Prior to fledging, males tended to be larger than females (*F*_1,11_ = 3.82, *p* = .08) while sex had no effect on chick condition (*F*_1,11_ < 0.01, *p* = .97).

### 3.3 Survival analysis

Mean weekly chick survival probability was 0.957 ± 0.011 (95% CI: 0.928–0.974). Model selection indicated that brooding duration did not affect the probability of death due to cat predation or predation by giant petrels (Table 3). Based on AICc values (ΔAICc = 3.71), there was some evidence that brooding duration was negatively related to the probability of death by flooding, although the 95% CI of the slope parameters overlapped with zero (slope = −4.358 ± 3.562; 95% CI: −11.340 to 2.625).

**TABLE 3:** Modeling the effects of brooding duration on wandering albatross chick survival and causes of death.

Model	Dev	Rk	AICc	ΔAICc	Slope
Reference model	154.4	5	164.6	0	
Effect on survival	153.6	6	165.8	1.2	−0.231 (−0.265 to 0.727
Death by cat predation	152.6	6	164.9	0.3	1.268 (−0.890 to 3.426)
Death by giant petrel predation	153.0	6	165.3	0.7	2.274 (−2.073 to 6.621)
Death by flooding	148.6	6	160.8	−3.8	−4.358 (−11.340 to 2.625)

Abbreviations: Dev, deviance; rk, model rank.

## 4 DISCUSSION

The aim of this study was to identify some potential determinants of brooding duration in wandering albatrosses and to question whether brooding duration is related to subsequent chick survival and biometry prior to fledging. Because the recent predation by feral cats has been shown to threaten wandering albatrosses Kerguelen population (Barbraud et al., 2021) and because extending brooding duration has been hypothesized as a parental tactic to face this new threat (Blanchard et al., 2024), we paid particular attention to cat predation risk. We set up a semi‐experimental design that led to an increased inter‐nests variance in feral cat abundance (Blanchard et al., 2024) and measured predator abundance at the nest scale using camera trap monitoring. Our results showed that brooding duration was independent of feral cat abundance, and also of giant petrel and skua abundance. Brooding duration depended on hatching date, with chicks hatched earlier in the season undergoing a longer brooding period. Our data also revealed that structural size at first capture was positively related to brooding duration. As capture occurred at 30–33 days for all chicks and brooding duration varied from 22 to 39 days, this relationship indicates that brooding had stopped by the time of capture for the smallest chicks and will continue after capture for the biggest chicks. We also showed that the frequency of parental shifts from hatching to capture positively impacted chick structural size at first capture and that parents were less inclined to end brooding in case of forthcoming unfavorable weather. Finally, brooding duration did not relate with subsequent chick survival nor with chick biometry prior to fledging, that is, at second capture, but tended to decrease the mortality risk associated with flooding.

Only few studies have investigated the role of predation risk at the nest scale on brooding duration (but see Catry et al., 2009; Varpe & Tveraa, 2005). Following the “chick‐protection hypothesis” (Catry et al., 2009), we predicted that chicks facing higher predation risk should benefit from a longer brooding and thus, protection period. However, our data clearly suggest that albatross parents did not use predator abundance as a cue over brooding duration decision, in line with previous results in Antarctic petrels (*Thalassoica antarctica*) (Varpe & Tveraa, 2005) and Cory's Shearwaters (*Calonectris diomedea*) (Catry et al., 2009). This may echo with the inability of pelagic seabirds to respond to the presence of introduced predators (Catry et al., 2009). If this inability stands from the recent appearance of these threats in their evolutionary history, such explanation could be particularly meaningful here for cats, as attacks by cats are probably recent (Blanchard et al., 2024). Moreover, cat attacks mostly occurred when parents were absent and many attacked chicks eventually died (Blanchard et al., 2024). This, together with the long generation time in wandering albatrosses, probably prevents a rapid emergence of a parental anti‐predator tactic, such as extending brooding duration. Alternatively, parents may rightly assess feral cats and giant petrels as a risk for their chick but may be unable to simultaneously increase brooding and provision their chicks often enough to sustain its growth. In addition, parents may also be reluctant to increase brooding duration because brooding is associated with a progressive decline in their body condition that cannot be sustained for several weeks without important survival costs (Weimerskirch & Lys, 2000). Blanchard et al. (2024) showed that chicks were at risk until at least 50 days old. Hence, even if every extra day of brooding is expected to be beneficial in terms of chick protection, ending brooding period when chicks are out of risk (i.e., extending brooding duration by 25%, for the longest brooding period we report, 39.2 days) may be too costly in terms of decrease in both parental condition and chick provisioning.

Our data revealed that parent wandering albatrosses were less prone to end brooding in case of unfavorable weather the following day. This is in line with the “cold protection hypothesis” (Catry et al., 2010, see also Weathers et al., 2000), suggesting that parents should respond to short‐term variations in weather, extending brooding period when facing bad weather to offer greater protection against cold, wind, or humidity, all parameters impacting chick survival in albatrosses (Catry et al., 2010; Cleeland et al., 2020; Lefebvre, 1977; Momberg et al., 2023). A previous study (Catry et al., 2010) also revealed the role of short‐term weather changes on black‐browed albatross parental decision to terminate brooding. Besides the expected effect of unfavorable weather on chick survival, parents may also be reluctant to end brooding and start long foraging trips under harsh weather as such conditions may impair their foraging success through reduced flight or detection ability (Nourani et al., 2023; Schreiber & Burger, 2001) and also possibly impact prey behavior (see Cabanellas‐Reboredo et al., 2012, for squids, an important prey of wandering albatrosses, Cherel et al., 2017). Finally, the proximate cue used by parents is presently unknown but may relate to changes in atmospheric pressure (Breuner et al., 2013; Metcalfe et al., 2013).

We also report a clear negative effect of hatching date on brooding duration, leading to an overall seasonal decline in brooding duration. This seasonal pattern was found in other birds (e.g., Rothenbach & Kelly, 2012), including seabirds (Catry et al., 2006, 2010; Pinto et al., 2016; Varpe & Tveraa, 2005), among which wandering albatrosses (Jones et al., 2014). The “synchronization hypothesis” (Catry et al., 2009) proposed that such pattern is triggered by a selective advantage for chicks reaching emancipation synchronously with other chicks because of a dilution and/or satiation effect of predators (Ims, 1990). Interestingly, Catry et al. (2009) reported no seasonal decline in brooding duration in a population of Cory's Shearwaters facing little predation, contrary to studies cited above, thereby further supporting this hypothesis. Here, however, we report a seasonal decline in brooding duration in a population facing low and recent predation risk (Blanchard et al., 2024) and where individual parents do not base their decision to end brooding on predator abundance. Hence, although such a seasonal decline may still be the result of an evolutionary history including predation risk pressure, other factors may be at work in our system (Catry et al., 2010; Moreno et al., 1997). Shorter brooding duration for late‐hatched chicks was suggested to be driven by a seasonal decline in food availability over the chick‐rearing period, triggering the necessity for late‐laying parents to resume foraging earlier in order to meet both the chick and their own energetic requirements (Catry et al., 2010). Indirect evidence suggests that wandering albatross prey availability may decline during chick‐rearing period (Salamolard & Weimerskirch, 1993), thereby offering a context for such selective pressure to occur in our system.

Non‐exclusively, hatching date may depend on parental quality, with high‐quality individuals, expected to be better able to face a longer fasting period (Tveraa et al., 1998; Tveraa & Christensen, 2002; Varpe et al., 2004; see also Weimerskirch & Lys, 2000 in wandering albatrosses) and to display better foraging skills/provisioning strategies (Lequette & Weimerskirch, 1990), nesting earlier in the season (Catry et al., 2010). Yet, several classical proxies of adult quality in seabirds (pair breeding experience, age, condition, and structural size) seem poorly related to hatching date in wandering albatrosses (Berrow et al., 2000; Jones et al., 2014; Lequette & Weimerskirch, 1990; Weimerskirch et al., 2000) so that this speculation deserves further investigation.

Finally, we report no effect of brooding duration on chick survival until fledging, contrary to a previous study on wandering albatrosses (Jones et al., 2014) but in line with results on grey‐headed albatrosses (Catry et al., 2006), Cory's shearwaters (Catry et al., 2009), or snow petrels (Dupont et al., 2023). Although a larger sample size would allow a more precise investigation of these questions, our results are not really surprising in our system. Given the identified sources of chick mortality for the study year and area, that is, ~72% by predation or nest flooding (Blanchard et al., 2024), and because cats preyed on chicks well after the end of the brooding period and irrespective of their mass (Blanchard et al., 2024), chick mortality is much more expected to depend on nest localization in respect to streams and home range of individual cats “specialized” on chicks (Blanchard et al., 2024) than to brooding duration. Still, increased brooding duration tended to reduce the probability of dying by flooding, perhaps because chicks with longer brooding duration hatched earlier, and were larger and thus less sensitive to flooding. However, this result should be interpreted with caution as our sample size was small, causing a low precision in the slope estimate. Exploring chick survival probability according to brooding duration in colonies not exposed to flooding and predation could lead to other conclusions.

## AUTHOR CONTRIBUTIONS

**Charlotte Bourgoin:** Formal analysis (equal); writing – original draft (equal). **Christophe Barbraud:** Conceptualization (supporting); data curation (supporting); funding acquisition (lead); methodology (supporting); project administration (equal); resources (lead); supervision (equal); writing – review and editing (supporting). **Tobie Getti:** Data curation (supporting). **Karine Delord:** Methodology (supporting); project administration (supporting); resources (supporting); writing – review and editing (supporting). **Frédéric Angelier:** Conceptualization (supporting); data curation (supporting); funding acquisition (supporting); resources (supporting); writing – review and editing (supporting). **Aymeric Bodin:** Conceptualization (supporting); funding acquisition (equal); methodology (supporting); project administration (supporting); resources (supporting). **Pierrick Blanchard:** Conceptualization (lead); data curation (lead); formal analysis (lead); methodology (lead); project administration (supporting); resources (equal); supervision (lead); writing – original draft (equal); writing – review and editing (lead).

## FUNDING INFORMATION

We thank the Réserve Naturelle Nationale des Terres Australes Françaises, the Institut Polaire Français Paul‐Emile Victor (IPF) (project number 109, PI C. Barbraud), and the Zone Atelier Antarctique et Terres Australes (CNRS‐INEE) for their fraternal, logistical, and financial support.

## CONFLICT OF INTEREST STATEMENT

The authors declare no conflict of interest.

## Data Availability

The datasets analyzed during the current study are available in the figshare repository, at https://figshare.com/s/75570849ccf548961ed8.
